# Supplementary figures and images for: Comparing statistical learning methods for complex trait prediction from gene expression
Source: bioRxiv. 2024 Jun 3:2024.06.01.596951. Preprint. [Version 1] doi: 10.1101/2024.06.01.596951 (PMC11185554; doi:10.1101/2024.06.01.596951)

A

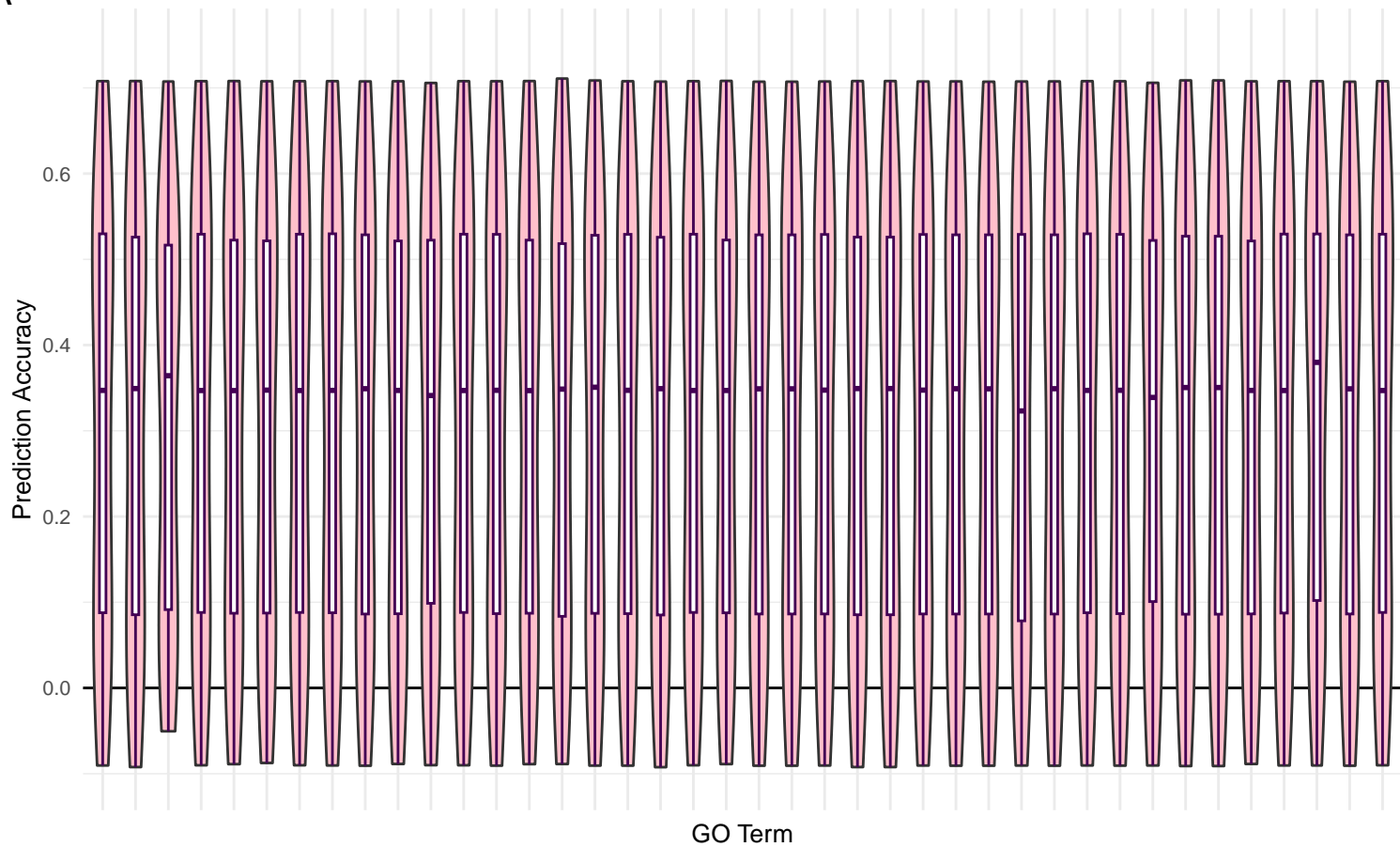

Supplement: Supplement 1 — S1 Fig. Violin plot comparison of Sparse Group Lasso results for top GO terms from GO-BayesC/GO-TBLUP along with randomly selected GO terms in females. [file media-1.pdf]

**A**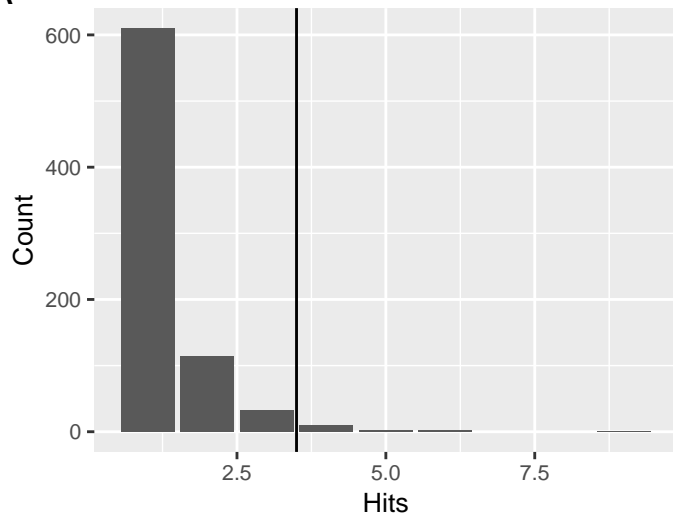**B**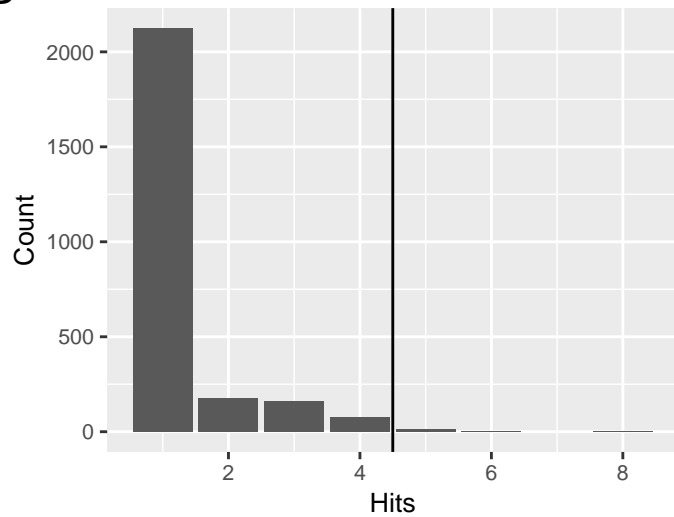**C**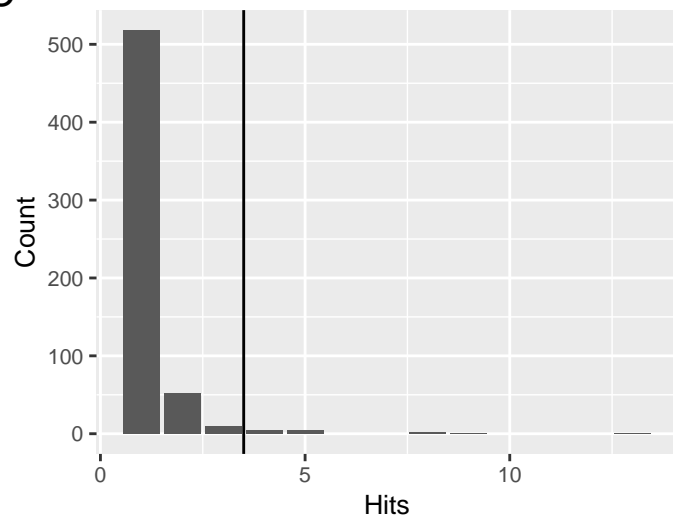**D**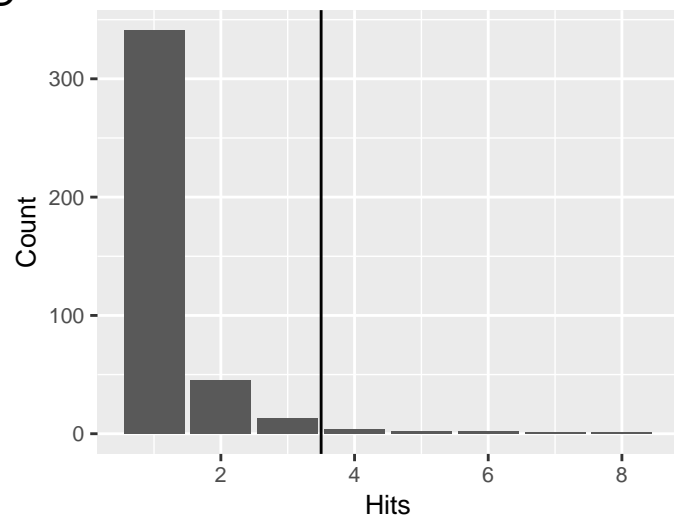

Supplement: Supplement 2 — S2 Fig. Distribution of number of overlapping genes for top 1% of GO terms for GO-BayesC and GO-TBLUP in females and males. The selection cutoff is marked by the vertical bar. [file media-2.pdf]
